# Accessory microbiomes

**DOI:** 10.1099/mic.0.001332

**Published:** 2023-05-11

**Authors:** Michiel Vos

**Affiliations:** ^1^​ European Centre for Environment and Human Health, Environment and Sustainability Institute, University of Exeter, Penryn Campus, TR10 9FE, Penryn, UK

**Keywords:** Microbiomes, accessory genomes, adaptation, dispersal

## Abstract

In microbiome research, considerable effort has been invested in finding core microbiomes, which have been hypothesized to contain the species most important for host function. Much less attention has been paid to microbiome members that are present in only a subset of hosts. Such accessory microbiomes must in large part consist of species that have no effect on fitness, but some will have deleterious effects on fitness (pathogens), and it is also possible that some accessory microbiome members benefit an ecologically distinct subset of hosts. This short paper discusses what we know about accessory microbiomes, specifically by comparing it with the concept of accessory genomes.

## Microbiomes

The term ‘microbiome’ can be taken as a synonym for ‘microbial community’ but is often more narrowly applied to microbial communities associated with hosts. Plants, animals, as well as single-celled algae and even multicellular bacteria [1] all come with an assemblage of associated microbes. The association of host and microbiome is often assumed to be mutually beneficial, with hosts benefitting through processes such as nutrient provision, protection against pathogens and modulation of the immune system, and microbes benefitting via nutrient provision and shelter [2]. Indeed, axenic (‘germ free’) mice that have their microbiomes artificially removed have impaired immune systems and show developmental defects [3], and it is challenging to grow many algal species in the absence of heterotrophic bacteria [4]. The interdependency of hosts and microbiomes has led to the concepts of the ‘hologenome’, the combined genomic information of host and their symbiotic microbiomes, and the ‘holobiont’, which treats the combined functional association as a single biological unit [5]. The tight association of microbial communities and their hosts is illustrated by the fact that phylogenetic relationships between host species are congruent with the ecological relationships of their microbial communities (‘phylosymbiosis’) [6].

## Core microbiomes

If microbiome members are crucial to host fitness, they can be expected to be present in all individual hosts. It is therefore no surprise that the search for specific functions mediating host health has focused on such ‘core microbiome’ members [7, 8]. However, core microbiomes have proven elusive, often representing only a very small number of the total microbiome. For instance, a recent study on kelp found that only five Amplicon Sequence Variants (ASVs) out of a total of 2824 ASVs were present in 95 % of samples (eleven ASVs were present in 80 % of samples) [9]. There are a variety of reasons that could make core microbiomes appear smaller than they are, from methodological, such as the specific taxonomic cut-offs used [10], to ecological, such as through microbiome variation with age, sex or season [11]. Moreover, as many microbiome functions could be redundant (i.e. carried out by more than a single species), it has been proposed that metagenomics sequencing quantifying specific genes or functions is superior to amplicon-based sequencing quantifying taxonomic diversity [12]. (Although this approach will still bring its own challenges, as it could be envisaged that a particular biological function could be performed by multiple distinct genes).

## Accessory microbiomes

The size of core microbiomes might be underestimated or really just very small, but it is clear that most microbiome members are not ‘core’, which means they must be ‘accessory’. Interestingly, the term ‘accessory microbiome’ is only sporadically mentioned in the literature. According to Vandenkoornhuyse *et al*., the accessory microbiome is expected to ‘contain more dispensable functions or micro-organisms whose presence is related to interactions with the surrounding environmental conditions’ [13]. However, this interpretation seems to disregard the possibility that some (or perhaps most) microbiome members might not interact with their host and instead are mere passengers. Perhaps the main way in which researchers have looked at a biologically meaningful variation in the distribution of microbiome members across hosts is in studies of microbiome markers of disease where the aim is to identify members of the microbiome, which are consistently present or absent in diseased versus healthy individuals. Indeed, part of the accessory microbiome will consist of pathogenic species present in diseased individuals only.

## Accessory genomes versus accessory microbiomes

There has been much recent interest in the concept of ‘pan genomes’ where strains belonging to the same species share a set of core genes, and with accessory genes being present in only a subset of strains [14]. This exactly mirrors the observation of ‘pan microbiomes’ where hosts belonging to the same species share a core microbiome (or at least are hypothesized to do so) with a much larger accessory microbiome present in many different subsets of hosts. Both fields study the patterns and causes of (not) sharing genes or microbial species, often visualized using Venn diagrams [8]. Whilst the microbiome literature has focused primarily on core microbiomes, the pangenome literature has tended to take the core genome for granted (presumably because it is obvious that a core of housekeeping genes must be present for a cell to function) and instead has directed its attention to the accessory genome. Regardless, both the community ecology processes shaping accessory microbiomes and population genetics processes shaping accessory genomes can be analysed in the same theoretical framework [15].

**Fig. 1.**
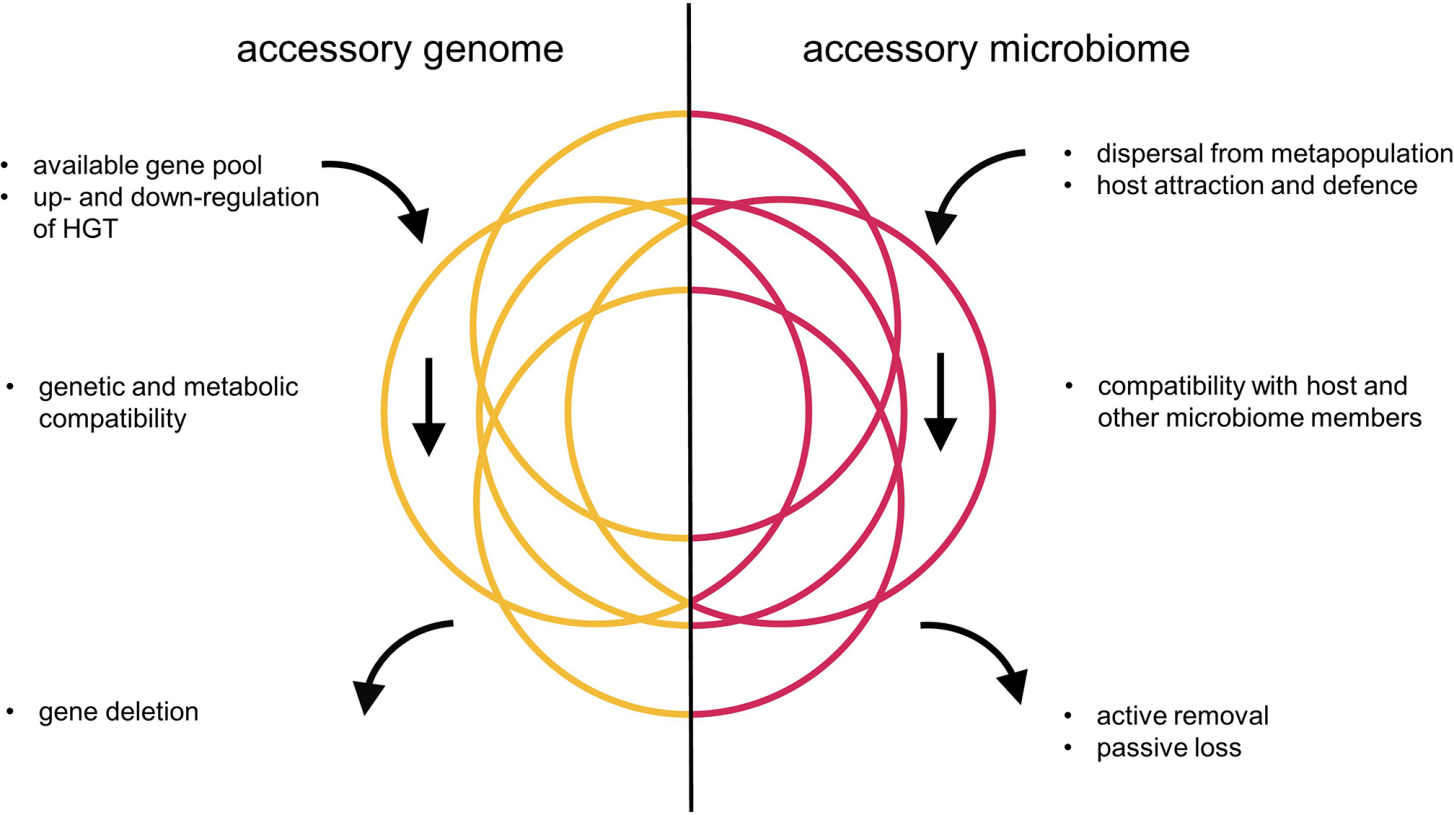
A simple diagram comparing the ecological and evolutionary forces that result in accessory genome and accessory microbiome variation. HGT=horizontal gene transfer.

Genomes as well as microbiomes experience random uptake: horizontal gene transfer (HGT) bringing in genes, and dispersal bringing in species (Fig. 1). Both these influxes can be shaped to some degree by the recipient: for instance, HGT can be downregulated through the acquisition of genetic barriers (e.g. restriction/modification systems) or upregulated through physiological responses (e.g. the development of competence for natural transformation). Microbiome species acquisition can be mediated by social behaviour [16] as well as host defences and the secretion of specific nutrients and attractants [2, 17]. New genes need to be genetically and metabolically compatible with other genes in the genome; new microbiome members likewise need to establish themselves in the face of inter-species competition. Some genes and species are infectious and detrimental to the fitness of their host. Genes are lost from genomes though a mutation-deletion bias if they are not retained by selection [18]. Microbiome members also face loss both through chance events, innate ephemerality (e.g. living in GI tracts) and active removal by the host or other microbiome members. Pan genomes can be open (large relative to the core genome) or closed (small relative to the core genome) and the same likely holds true for pan microbiomes. It will be interesting to see whether large pan microbiome sizes are mainly due to increased colonization rate or retention rate (or both).

## Outlook

**Fig. 2.**
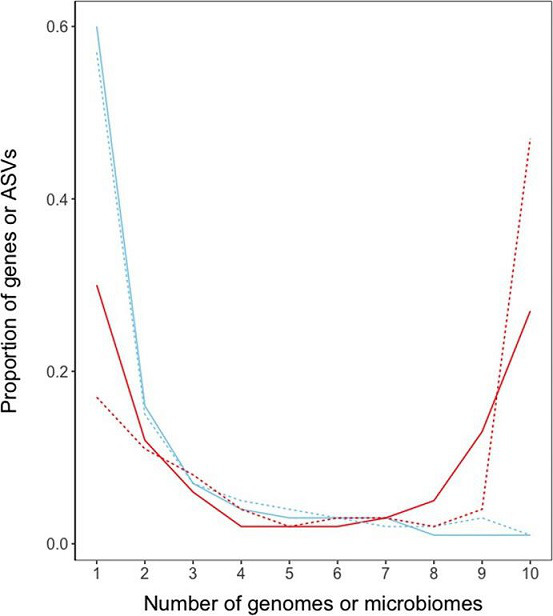
Frequency distribution of genes in the pan genomes (red) of *Escherichia coli* (solid line) and *Staphylococcus aureus* (dotted line) versus amplicon sequence variants (‘species’ or ‘genotypes’) (blue) in kelp (solid line) and roach (dotted line) ‘pan microbiomes’. Presence/absence of each gene or ASV was scored in each of ten genomes or ten microbiomes. Data from [9, 24, 25].

To what extent accessory genomes are made up of genes involved in adaptation or genes that have neutral or even deleterious fitness effects is still under debate [19]. It seems that this same debate has not yet started in the case of accessory microbiomes. To find out whether an appreciable part of the accessory microbiome consists of species that provide benefits specific to host ecotypes, or that most microbiome members are simply present due to random chance, new datasets and analyses are needed. Sequencing microbiomes from ecologically distinct populations within a species could help define functionally distinct accessory microbiomes, akin to clade-specific accessory genomes [20]. However, identifying functionally relevant accessory microbiome members is not straightforward, as different host ecotypes that live in different niches will also differentially encounter, and be colonized by, species that have no positive effect on fitness. Laboratory experiments using tractable model systems where microbiome members could be added and removed and host fitness reliably quantified [21] could minimize ecological stochasticity. Community ecology modelling, including network analysis, could shed light on the distribution of different taxa among a population of hosts [22]. Accessory genomes have a distinctive U-shaped distribution, with many accessory genes being rare or abundant, and only few being of intermediate frequency [23], which is not the case for accessory microbiomes (Fig. 2). This could indicate that selection operates differently on accessory microbiomes than it does on accessory genomes (although it must be noted that genes are generally present in a single copy per genome, whereas the abundance of amplified ASVs ranges from one to thousands per microbiome sample, which complicates this comparison). It is hoped a variety of approaches will be used to answer the question whether the small size of core microbiomes is in part due to the distribution of host-relevant microbes across ecologically distinct specialists.
